# Interleukin‐6 as a Key Biomarker in Facioscapulohumeral Dystrophy: Evidence From Longitudinal Analyses

**DOI:** 10.1002/acn3.70210

**Published:** 2025-10-07

**Authors:** Jonathan Pini, Emanuela Martinuzzi, Sandra Dhifallah, Abderhmane Slioui, Angela Puma, Luisa Villa, Michele Cavalli, Andra Ezaru, Jérémy Garcia, Manuela Gambella, Federico Torre, Luca Jacopo Pavan, Nicolas Glaichenhaus, Sabrina Sacconi

**Affiliations:** ^1^ Peripheral Nervous System and Muscle Department Nice University Hospital, Pasteur 2 Hospital Nice France; ^2^ Institute of Molecular and Cellular Pharmacology University Cote d'Azur, CNRS, Inserm Valbonne France; ^3^ Princess Grace Hospital Center Monaco Monaco; ^4^ Department of Diagnostic and Interventional Radiology Nice University Hospital, Pasteur 2 Hospital Nice France; ^5^ University Cote d'Azur, Inserm, CNRS, Institute for Research on Cancer and Aging of Nice (IRCAN) Nice France

## Abstract

**Objective:**

Facioscapulohumeral muscular dystrophy type 1 (FSHD1) is a progressive neuromuscular disorder with no approved treatments. Identifying reliable biomarkers is critical to monitor disease severity, activity, and progression. Interleukin‐6 (IL‐6) has been proposed as a candidate biomarker, but longitudinal validation is limited.

**Methods:**

We analyzed pooled data from two prospective longitudinal cohorts: CTRN‐FSHD France (NCT04038138) and Cytokine FSHD (NCT04694456), each comprising 30 genetically confirmed ambulant FSHD1 patients. Serum IL‐6 levels and clinical assessments were collected at baseline (M0), 12 months (M12), and 18 months (M18); whole‐body muscle MRI (T1‐weighted and STIR sequences) was obtained at M0 and M12. Associations between IL‐6 levels and clinical severity scores, functional measures, and MRI‐derived muscle composition were evaluated.

**Results:**

Serum IL‐6 levels correlated significantly with clinical severity metrics, including Clinical Severity Score, 6‐Minute Walk Test, Manual Muscle Testing, and Motor Function Measure Domain 1 at all time points. Higher IL‐6 levels were associated with increased muscle fat infiltration and free water content compatible with muscle edema on MRI. Longitudinal analyses showed that increases in IL‐6 over 12 months were significantly correlated with changes in T1 (fat infiltration) and STIR (muscle edema) composite scores, reflecting structural and inflammatory disease progression.

**Interpretation:**

These findings validate IL‐6 as a biomarker of FSHD1 severity and underscore its potential as an activity and progression biomarker. The correlation between IL‐6, clinical scores, and MRI‐based muscle composition changes highlights its potential utility for monitoring disease evolution and evaluating therapeutic responses in FSHD1 patients.

**Abbreviations**
6MWT: 6‐Minute Walk Test
CSS: clinical severity score
DUX4: double homeobox 4
FOV: field of view
FSHD: facioscapulohumeral dystrophy
FSHD1: facioscapulohumeral dystrophy type 1
IL‐6: interleukin‐6
IL‐10: interleukin‐10
LLOD: lower limit of detection
MFM‐D1: domain 1 of the motor function measure
MMT: manual muscle testing
MRI: magnetic resonance imaging
MSD: meso scale discovery
Nb: number
RUs: repeat units
STIR: short tau inversion recovery
T1‐W: T1‐weighted imaging
TE: echo time
TI: inversion time
TNF‐α: tumor necrosis factor alpha
TR: repetition time
TSE: turbo spin echo

## 1 Introduction

Facioscapulohumeral muscular dystrophy (FSHD) is a common adult muscular dystrophy, affecting 2–7 per 100,000 individuals [1, 2], causing progressive facial, scapular, and humeral muscle weakness with highly variable presentation [3, 4]. FSHD1, the most common form, results from D4Z4 repeat contraction on chromosome 4q35, leading to pathological *DUX4* expression [5, 6]. This drives muscle degeneration and atrophy, further exacerbated by inflammation [7, 8].

While *DUX4* activation is central to FSHD, immune activation and inflammation are increasingly recognized as key drivers of disease progression [7, 9]. FSHD1 muscle inflammation involves immune cell infiltration and dysregulated cytokine production, including IL‐6, TNF‐α, and IL‐10 [10]. MRI studies show that inflammation precedes muscle degeneration and fibrosis, worsening disease severity [11, 12]. However, the interplay between genetic and inflammatory pathways remains unclear, highlighting the need for further research to identify reliable biomarkers.

Recent advances in the study of FSHD1 biomarkers have highlighted serum interleukin‐6 (IL‐6) as a particularly promising candidate [13, 14]. Elevated serum IL‐6 levels have been observed in various neuromuscular disorders, correlating with disease severity and progression [15, 16]. When chronically secreted, IL‐6 has essentially a proinflammatory and catabolic effect on muscle, promoting muscle wasting and inhibiting muscle regeneration [17]. However, its precise role in FSHD1 remains under investigation, with questions surrounding its origin and its importance for FSHD1 pathophysiology.

Muscle Magnetic Resonance Imaging (MRI) has also emerged as an imaging biomarker in neuromuscular diseases [18] and is a secondary endpoint of several clinical trials in FSHD1 [19, 20].

T1‐weighted imaging can semi‐quantify muscle fat infiltration on a four‐point scale, correlating with disease activity [21]. Fat‐saturated sequences, typically STIR hyperintensity, are commonly interpreted as muscle edema linked to inflammation in various myopathies [18, 22, 23]. In FSHD1, STIR changes align with biopsy findings and inflammatory gene upregulation, indicating muscle edema as a marker of disease activity [24]. Changes in muscle edema, associated with inflammation and fibro‐fatty infiltration, precede clinical deterioration, as evidenced by MRI abnormalities detected even in asymptomatic FSHD1 patients [25].

The present study integrates data from two independent longitudinal cohorts of 60 ambulant adult FSHD1 patients to achieve three key objectives. First, we aimed to confirm IL‐6 as a biomarker of disease severity by assessing its correlation with clinical scores at multiple time points (Baseline, 12 months, and 18 months) and muscle MRI T1 Sum Score (Baseline and 12 months). Second, we explored IL‐6 as a biomarker of disease activity by evaluating its association with muscle MRI STIR Sum Score, commonly associated with inflammation and edema at two time points (Baseline and 12 months). Third, we examined IL‐6 as a potential biomarker of disease progression by analyzing its dynamic relationship with changes in muscle MRI composite STIR and T1 Sum Scores, alongside variations in clinical scores over time.

Our findings confirm serum IL‐6 as a robust biomarker of FSHD1 severity, with moderate correlations to clinical severity, functional performance, and MRI markers of muscle pathology. Furthermore, our findings show that IL‐6 levels correlate with disease progression, underscoring its potential as a biomarker of disease activity and progression for monitoring disease activity and evaluating therapeutic interventions.

## 2 Methods

### 2.1 Patient Study Design and Participants

The Cytokines FSHD (NCT04694456) and CTRN‐FSHD France (NCT04038138) studies were prospective, monocentric, 18‐month trials, each enrolling 30 FSHD1 patients. Studies spanned from January 2018 to April 2021 for Cytokines FSHD, and from September 2019 to September 2021 for CTRN‐FSHD France. All participants provided written informed consent.

### 2.2 Study Population

All patients were referred to the Neuromuscular Department at Nice University Hospital, France, and met specific inclusion and exclusion criteria.

Inclusion criteria were: genetically confirmed FSHD1 (D4Z4 repeat < 11 with a permissive 4qA allele) or clinical diagnosis with family history; age 18–75 years; symptomatic limb weakness; ability to walk independently or with aid; MMT score ≥ 4 in at least one lower limb muscle; and stable use of over‐the‐counter supplements.

Exclusion criteria included: recent exposure to vaccines, blood products, or tropical residence; use of immunosuppressive/immunomodulatory drugs; HIV, HBV, or HCV seropositivity; recent infections; chronic diseases affecting immune response; substance abuse; unstable cardiac or respiratory conditions; orthopedic limitations; regular use of muscle anabolic/catabolic agents; recent malignancy; recent intense exercise; MRI contraindications; special diet regimens; or participation in other investigational studies.

### 2.3 Collected Data

For all patients, data collected included D4Z4 repeat size, age at examination and disease onset, disease duration, comorbidities, and current and past therapies. Clinical Severity Score (CSS) and age‐corrected CSS were recorded as previously described [26]. Motor function was assessed by Manual Muscle Testing (MMT) sum score across 28 muscle groups and Domain 1 of the Motor Function Measure (MFM‐D1), which evaluates standing and transfers through 13 items [27]. Mobility and balance were assessed with the 6‐Minute Walk Test (6MWT), a validated measure of functional exercise capacity widely used in neuromuscular disease studies [28].

### 2.4 Muscle Magnetic Resonance Imaging (MRI)

Whole‐body muscle MRI was performed using a 1.5 Tesla scanner (Magnetom Espree, Siemens AG) with integrated phased‐array coils. Non‐contrast axial, coronal, and sagittal images were acquired using T1‐TSE sequences, and axial fat‐suppressed STIR sequences were used to assess muscle edema. T1 axial imaging (TR/TE 400/13 ms) was obtained in 2–3 contiguous stacks covering from the skull base to T10 vertebrae. STIR axial images (TR/TE/TI 3000/35/160 ms) matched the T1 geometry. Coronal and sagittal T1 images were acquired with specific orientations to optimize visualization of anterior thoracic, posterior dorsal, neck, and lower limb muscles. Each MRI session lasted approximately 45 min.

A total of 94 muscles on both sides were scored for each patient (14 axial, 26 upper limb, 54 lower limb). Pseudonymized images were independently scored by two experienced radiologists blinded to acquisition time, clinical, and IL‐6 data, with consensus reached for discordant cases (> 80% agreement overall). The complete list of the evaluated muscles is available in Table S1.

Based on T1‐W or STIR images, muscles were evaluated on all available sections. Fat infiltration was graded into five classes based on percentage involvement [21], while muscle edema was categorized into 2 distinct classes based on the presence or absence of STIR hyperintense signal. The detailed MRI muscle classes are shown in Table S2.

The overall average fat infiltration across all muscles was calculated using a weighted formula:

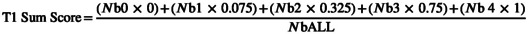

where *N*b0, number of muscles classified as Class 0; *N*b1, number of muscles classified as Class 1; *N*b2, number of muscles classified as Class 2; *N*b3, number of muscles classified as Class 3; *N*b4, number of muscles classified as Class 4; *N*bALL, total number of muscles analyzed (94).

The STIR Sum Score was calculated as the proportion of STIR‐positive muscles among the 94 evaluated, as follows:

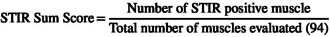

Representative STIR and T1‐weighted images and the associated classes are shown in Figure S1a,b, respectively.

### 2.5 Patient Serum Samples

Patients' serum samples were obtained and processed as previously described [13].

### 2.6 Immunoassay for Patient Samples

Serum IL‐6 levels were measured using the ultrasensitive S‐PLEX kit (Meso Scale Discovery, Rockville, MD) following the manufacturer's instructions. Samples were processed in batches of 80 alongside calibrated standards. Data were acquired using the V‐PLEX Sector Imager 2400 and analyzed with Discovery Workbench 3.0 software. Standard curves were generated from serial 2‐fold dilutions of premixed lyophilized standards, with diluent alone as a blank. IL‐6 concentrations were extrapolated using a 4‐parameter logistic curve. The lower limit of detection (LLOD) was 0.025 pg/mL, defined as a signal ≥ 2.5 standard deviations above the blank (zero calibrator).

### 2.7 Statistical Analysis

No outliers were detected using the GraphPad Prism 8.2.1 ROUT algorithm with the most stringent parameter (*Q* = 0.1%). Correlations between serum IL‐6 levels and clinical and paraclinical scores were computed using with Pearson's correlation coefficient when variables were normally distributed or Spearman's rank correlation coefficient otherwise with Benjamini & Hochbergs multiple test correction (False Discovery Rate, FDR at 5%). Normality was assessed with the Shapiro–Wilk test. Paired two‐tailed t‐tests were used for two sample comparisons. Statistical analyses were performed using GraphPad Prism 8.2.1. Statistical significance was evaluated according to the following *p*‐values: *p < 0.05, **p < 0.01, ***p < 0.001, ****p < 0.0001.

## 3 Results

### 3.1 Study Designs

The Cytokines FSHD study (NCT04694456) and the CTRN‐FSHD France study (NCT04038138) were designed as two independent prospective, monocentric, 18‐month studies with a similar protocol. Both studies included 30 FSHD1 patients and shared three common time points (Baseline M0), 12‐month (M12), and 18‐month (M18) follow‐up visits. Participants underwent a single‐day evaluation at each visit, following the schedule of activities outlined in Table S3. To increase statistical power, we performed a pooled data analysis by pooling data from both the Cytokines FSHD and CTRN‐FSHD France studies.

### 3.2 Patient Demographical and Clinical Data

Of the 60 patients enrolled, 52 completed the 12‐ and 18‐month follow‐up, forming our final cohort for analysis. Over time, patients showed progressive clinical deterioration, with worsening Clinical Severity Scores (CSS), decreased muscle strength (MMT), reduced motor function (MFM‐D1), and lower 6‐Minute Walk Test (6MWT) distances. Muscle MRI revealed increasing fat infiltration (T1 Sum Score) and muscle edema (STIR Sum Score), confirming ongoing structural degeneration and inflammation. No significant differences were observed between 12‐ and 18‐month follow‐ups between males and females across demographic or clinical variables. Detailed values at the Baseline, 12‐month, and 18‐month visits, and the *p*‐values, are presented in Table 1.

**TABLE 1:** Patient demographic and clinical characteristics.

	Baseline (M0)	12‐month visit (M12)	18‐month visit (M18)	*T*‐test *p* (M12‐M0)	*T*‐test *p* (M18‐M0)
Age at examination	53.29 ± 15.02	54.29 ± 15.02	54.42 ± 15.09	/	/
Age at disease onset	32.77 ± 16.07 (From 10 to 70)			/	/
Disease duration	20.52 ± 14.38	21.52 ± 14.38	21.65 ± 14.45	/	/
4qA D4Z4 RUs	6.62 ± 0.23 (From 3 to 10)			/	/
CSS	5.44 ± 2.12 (from 2 to 8)	6.17 ± 1.69 (From 2 to 9)	6.07 ± 1.76 (From 2 to 9)	*p* = 0.0004	*p* = 0.0018
Age‐corrected CSS	109.13 ± 52.06 (from 28.2 to 241.1)	122.13 ± 48.78 (From 34.5 to 296.3)	119.16 ± 48.46 (From 34.48 to 296.3)	*p* = 0.006	*p* = 0.013
MMT sum score	114.65 ± 17.61 (from 69.33 to 140)	110.86 ± 18.63 (From 71.33 to 138)	110.96 ± 19.87 (From 65.28 to 139.7)	*p* < 0.0001	*p* = 0.006
MFM‐D1	31.06 ± 8.85 (from 7 to 39)	28.31 ± 9.75 (From 5 to 39)	28.63 ± 10.03 (From 4 to 35)	*p* = 0.0004	*p* = 0.0005
6MWT	464.73 ± 168.5 (from 129 to 750)	397.62 ± 173.6 (From 72 to 740)	411.35 ± 179.8 (From 72 to 690)	*p* < 0.0001	*p* = 0.0006
T1 sum score	0.20 ± 0.17 (from 0 to 0.58)	0.26 ± 0.20 (From 0 to 0.68)	/	*p* < 0.0001	/
STIR sum score	0.06 ± 0.06 (from 0 to 0.22)	0.14 ± 0.12 (From 0 to 0.59)	/	*p* < 0.0001	/
IL‐6 (pg/mL)	53.93 ± 42.74 (from 8.03 to 283.81)	125.18 ± 117.1 (from 14.45 to 707.84)	88.81 ± 67.9 (from 13.80 to 364.4)	*p* < 0.0001	*p* < 0.0001
Log (IL6)	1.63 ± 0.30 (from 0.9 to 2.45)	1.97 ± 0.33 (From 1.16 to 2.85)	1.85 ± 0.19 (From 1.14 to 2.56)	*p* < 0.0001	*p* < 0.0001

*Note:* Data expressed as means ± SD.

Abbreviations: 6MWT, 6 Minute Walk Test; CSS, clinical severity score; MFM, motor function measure; MMT, manual muscle testing; RUs, repeat units.

All clinical scores demonstrated statistically significant differences between baseline and the 12‐month visit and baseline and the 18‐month visit, indicating that FSHD1 patients included in this study experienced a deterioration in muscle function, mobility, and muscle composition over the course of 12 and 18 months. No differences were observed between the 12‐ and 18‐month visits.

### 3.3 MRI Muscle Fat Content Is Associated With Disease Severity

To assess the association between intramuscular fat and disease severity, we analyzed correlations between the T1 Sum Score, CSS, and functional measures at baseline and 12 months (the only time points with MRI data in the Cytokines FSHD study). The comparison between baseline and the 12‐month visit, along with the changes in individuals over time in T1 Sum Score is represented in Figure S6a,b, respectively.

At both time points, higher T1 Sum Scores were significantly associated with longer disease duration (*r* = 0.52, *p* < 0.0001, Figure S2a for baseline and *r* = 0.54, *p* < 0.0001, Figure S3a for 12 months), greater clinical severity (*r* = 0.66, *p* < 0.0001 for CSS, and *r* = 0.42, *p* = 0.002 for age‐corrected CSS, Figure S2b,c for baseline and *r* = 0.70, *p* < 0.0001 for CSS, and *r* = 0.33, *p* = 0.002 for age‐corrected CSS, Figure S3b,c for 12 months), and poorer functional performance, including reduced MMT scores (Figure S2d for baseline and Figure S3d for 12 months), shorter 6MWT distances (*r* = −0.65, *p* < 0.0001, Figure S2e for baseline and *r* = −0.65, *p* < 0.0001, Figure S3e for 12 months), and lower MFM‐D1 scores (Figure S2f for baseline and Figure S3f for 12 months). Notably, the strongest associations were observed with MFM‐D1 (*r* = −0.85 at baseline; *r* = −0.84 at 12 months, both with *p* < 0.0001) and MMT (*r* = −0.79 at baseline; *r* = −0.78 at 12 months, both with *p* < 0.0001), indicating a robust link between fat infiltration and motor decline. These associations remained statistically significant after adjusting for age (Table S4). These findings indicate that higher intramuscular fat content, reflected by elevated T1 Sum Scores, is strongly associated with greater disease severity in FSHD1.

### 3.4 MRI Muscle Edema Is Associated With Disease Activity

To determine whether muscle edema reflects disease activity in FSHD1, we examined correlations between the STIR Sum Score, T1 Sum Score, CSS, and functional measures at baseline and 12 months. The comparison between Baseline and the 12‐month visit, along with the changes in individuals over time in STIR Sum Score, is represented in Figure S6c,d, respectively. At baseline, STIR Sum Scores showed moderate positive correlations with disease duration (*r* = 0.39, *p* = 0.004, Figure S4a), clinical severity (*r* = 0.27, *p* = 0.028, Figure S4b), and T1 Sum Scores (*r* = 0.38, *p* = 0.0048, Figure S4g), and a negative correlation with motor function (MFM‐D1, *r* = −0.37; *p* = 0.008, Figure S4f). After adjusting for age, associations with age‐corrected CSS, MFM‐D1, and T1 Sum Scores remained significant (Table S4).

At 12 months, stronger correlations emerged: STIR Sum Scores were significantly associated with disease duration (*r* = 0.38, *p* = 0.006, Figure S5a), clinical severity (*r* = 0.48, *p* = 0.0001, Figure S5b), T1 Sum Scores (*r* = 0.62, *p* < 0.0001, Figure S5g), and functional decline, including both MMT (*r* = −0.38, *p* = 0.006, Figure S5d) and MFM‐D1 scores (*r* = −0.44, *p* = 0.001, Figure S5f). These associations also held in age‐adjusted analyses (Table S4). Notably, the strongest and most consistent relationship was observed between STIR and T1 Sum Scores, supporting a link between muscle inflammation and structural degeneration. These findings reinforce the role of STIR hyperintensities as markers of active disease and predictors of progression toward fatty infiltration and muscle degradation [12, 29, 30].

### 3.5 Muscle MRI Metrics Are Associated With Disease Progression

To assess whether MRI changes reflect disease progression, we analyzed correlations between changes in STIR and T1 Sum Scores and changes in clinical outcomes over time. No significant associations were observed between MRI changes and changes in CSS, MMT, 6MWT, or MFM‐D1, possibly reflecting limited variability in these clinical measures. However, a significant correlation was found between changes in STIR and T1 Sum Scores (*r* = 0.47, *p* = 0.0001, Figure 4a), indicating that increased muscle inflammation is associated with progressive fat infiltration. This suggests that MRI‐based measures may capture early pathological changes not yet reflected in functional decline.

### 3.6 Circulating IL‐6 Is a Biomarker of Disease Severity

Serum IL‐6 levels were assessed at baseline, 12 months, and 18 months in the final FSHD1 cohort (*n* = 52). Levels increased significantly over time, from 53.4 ± 42.74 pg/mL at baseline to 125.2 ± 117.1 pg/mL at 12 months, followed by a partial decline to 88.8 ± 67.9 pg/mL at 18 months (both with *p* < 0.0001 vs. baseline), though values remained elevated (Table 1 and Figure S6e). Change in individuals over time is represented in Figure S6f. This temporal pattern paralleled progressive clinical deterioration observed in functional and severity scores.

**FIGURE 1:**
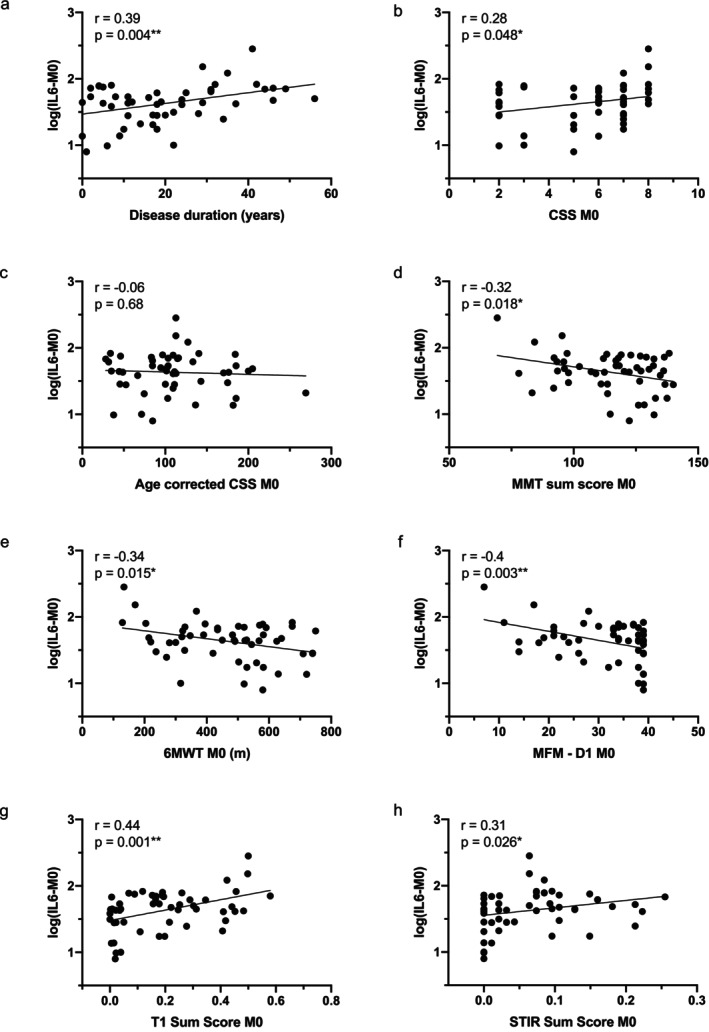
Correlation between serum IL‐6 levels and clinical, functional, and MRI‐derived score at baseline. Scatter plot representations of serum IL‐6 levels versus disease duration (a), clinical severity score (CSS; b), age‐corrected CSS (c), Manual Muscle Testing (MMT) sum score (d), 6 Minute Walk Test (6MWT; e), Domain 1 of the Motor Function Measure (MFM‐D1; f), T1 Sum Score (g), and the STIR Sum Score (h) at baseline. Spearman or Pearson *r* coefficients and *p*‐values are indicated (*p < 0.05, **p < 0.01).

At baseline, higher IL‐6 levels correlated positively with disease duration (*r* = 0.39, *p* = 0.004, Figure 1a), clinical severity (CSS; *r* = 0.28, *p* = 0.048, Figure 1b), and muscle fat infiltration (T1 Sum Score; *r* = 0.44, *p* = 0.001, Figure 1g), and negatively with MMT (*r* = −0.32, *p* = 0.018, Figure 1d), 6MWT (*r* = −0.34, *p* = 0.015, Figure 1e), and MFM‐D1 (*r* = −0.40, *p* = 0.003, Figure 1f). These associations remained significant after adjusting for age (Table S5). No correlation was observed with the age‐corrected CSS.

**FIGURE 2:**
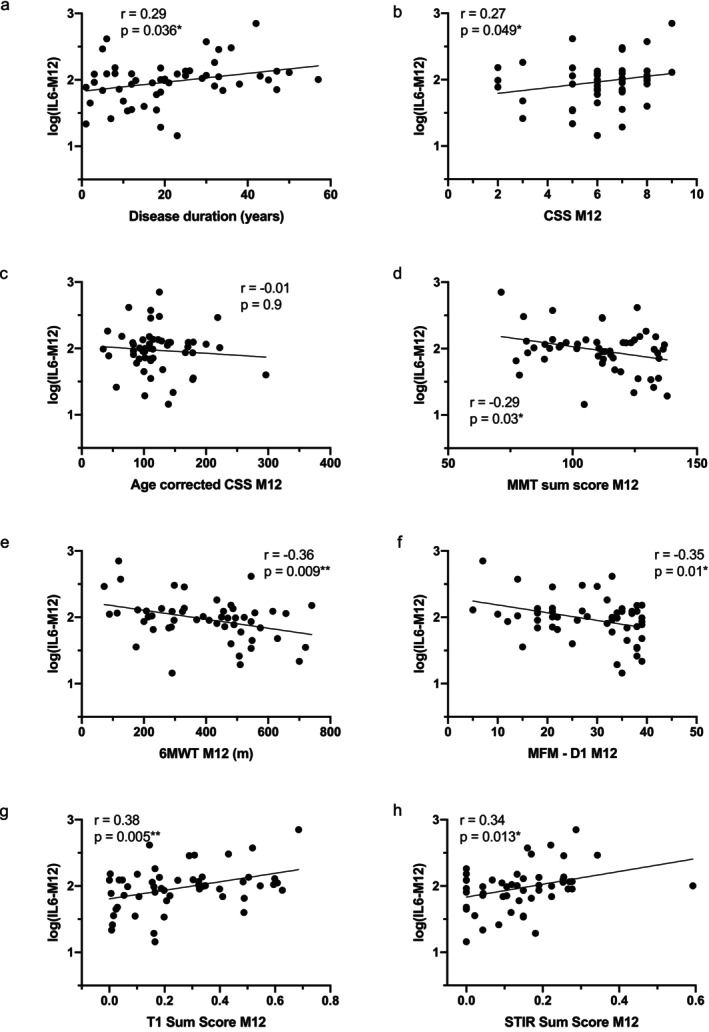
Correlation between serum IL‐6 levels and clinical, functional, and MRI‐derived score at the 12‐month visit. Scatter plot representations of serum IL‐6 levels versus disease duration (a), clinical severity score (CSS; b), age‐corrected CSS (c), Manual Muscle Testing (MMT) sum score (d), 6 Minute Walk Test (6MWT; e), Domain 1 of the Motor Function Measure (MFM‐D1; f), T1 Sum Score (g), and the STIR Sum Score (h) at the 12‐month visit. Spearman or Pearson r coefficients and *p*‐values are indicated (*p < 0.05, **p < 0.01).

At 12 months, these trends persisted. IL‐6 remained significantly associated with disease duration (*r* = 0.29, *p* = 0.036, Figure 2a), CSS (*r* = 0.27, *p* = 0.049, Figure 2b), T1 Sum Score (*r* = 0.38, *p* = 0.005, Figure 2g), and negatively with MMT (*r* = −0.29, *p* = 0.03, Figure 2d), 6MWT (*r* = −0.36, *p* = 0.009, Figure 2e), and MFM‐D1 (*r* = −0.35, *p* = 0.01, Figure 2f). Correlations remained significant after age adjustment, while age‐corrected CSS remained non‐significant (Table S5).

**FIGURE 3:**
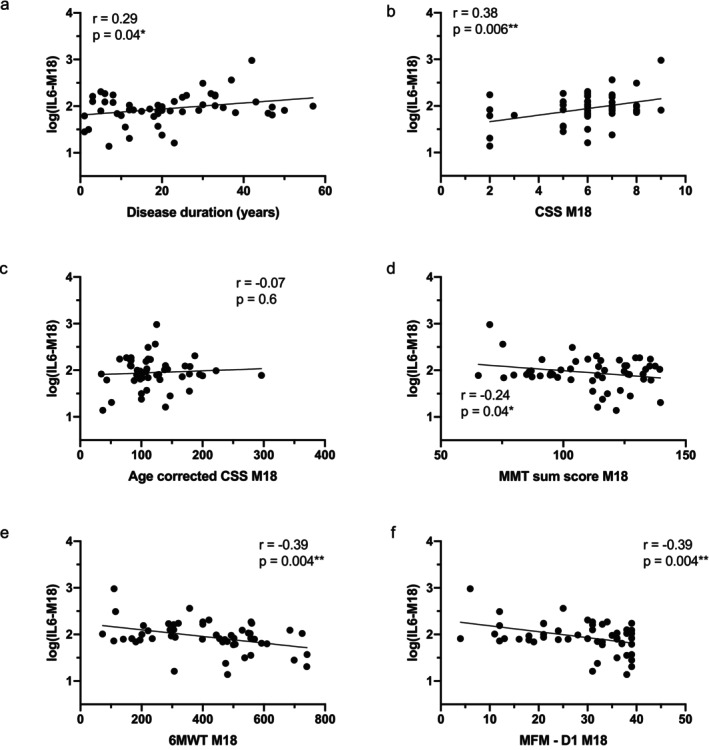
Correlation between serum IL‐6 levels and clinical score at the 18‐month visit. Scatter plot representations of serum IL‐6 levels versus disease duration (a), clinical severity score (CSS; b), age‐corrected CSS (c), Manual Muscle Testing (MMT) sum score (d), 6 Minute Walk Test (6MWT; e), and Domain 1 of the Motor Function Measure (MFM‐D1; f), at the 18‐month visit. Spearman or Pearson *r* coefficients and *p*‐values are indicated (*p < 0.05, **p < 0.01).

At 18 months, IL‐6 continued to correlate with disease duration (*r* = 0.34, *p* = 0.01, Figure 3a), CSS (*r* = 0.42, *p* = 0.001, Figure 3b), and functional decline (MMT: *r* = −0.30, *p* = 0.02, Figure 3d; 6MWT: *r* = −0.40, *p* = 0.003, Figure 3e; MFM‐D1: *r* = −0.41, *p* = 0.002, Figure 3f). After adjusting for age, these correlations persist. Although unadjusted associations with age‐corrected CSS remained non‐significant, age‐adjusted analyses revealed a moderate correlation (Table S5).

Together, these findings confirm that elevated serum IL‐6 is consistently associated with greater disease severity, reduced motor function, and increased fat infiltration over time. The robustness of these associations across multiple time points and after age adjustment supports IL‐6 as a relevant biomarker of disease severity in FSHD1.

### 3.7 Circulating IL‐6 Is a Potential Biomarker of Disease Activity

To explore whether serum IL‐6 levels reflect disease activity, we analyzed their association with the STIR Sum Score, a marker of muscle edema associated with inflammation, at baseline and after 12 months.

At baseline, IL‐6 levels were positively correlated with STIR Sum Score (*r* = 0.31, *p* = 0.026, Figure 1h), and the association remained significant after adjusting for age (Table S5). This relationship persisted at 12 months, with IL‐6 again showing a significant correlation with STIR Sum Score (*r* = 0.34, *p* = 0.013, Figure 2h), which remained robust following age adjustment (Table S5). These findings indicate that higher serum IL‐6 levels are associated with greater muscle inflammation, supporting IL‐6 as a potential biomarker of disease activity in FSHD1.

### 3.8 Circulating IL‐6 Is a Potential Biomarker of Disease Progression

**FIGURE 4:**
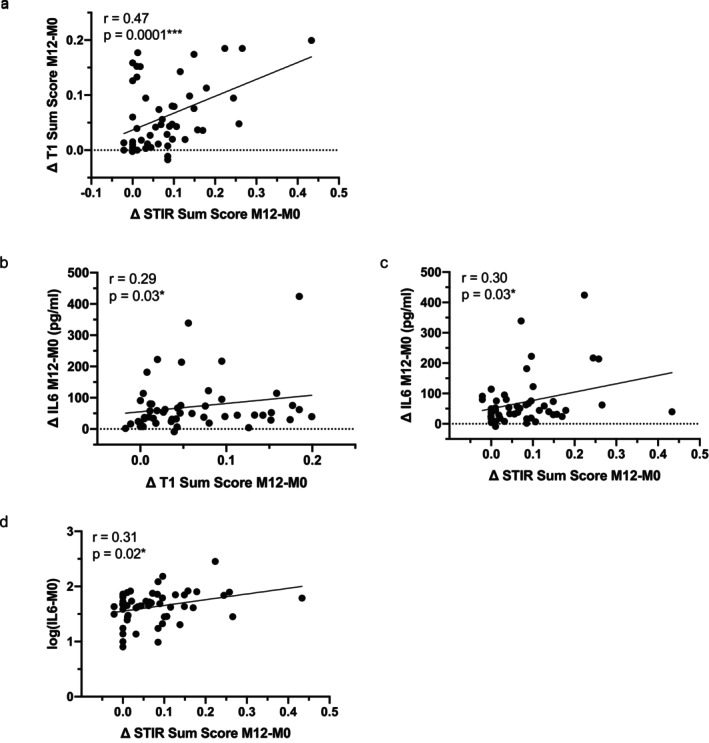
Correlations between change from Baseline serum in MRI‐Composite score, clinical scores, and IL‐6 levels over 12 and 18 months. Scatter plot representations of the change from baseline in the T1 Sum Score versus change from baseline in the STIR Sum Score (a), and the change from baseline in serum IL‐6 levels versus the change from baseline in T1 Sum Score (b), the STIR Sum Score (c), and serum IL‐6 levels versus change from baseline in the STIR Sum Score (d). Spearman or Pearson *r* coefficients and *p*‐values are indicated (*p < 0.05, ***p < 0.001).

Given the consistent associations between IL‐6 levels, clinical measures, and MRI findings across all time points, we examined whether changes in IL‐6 levels could reflect disease progression. While no significant correlations were found between changes in IL‐6 and clinical scores over 12 or 18 months, imaging analyses revealed otherwise. Over the 12‐month period, changes in IL‐6 levels were positively correlated with changes in both the T1 Sum Score (*r* = 0.29, *p* = 0.03; Figure 4b) and STIR Sum Score (*r* = 0.30, *p* = 0.03; Figure 4c), indicating that rising IL‐6 levels were associated with increased fat infiltration and muscle edema, respectively. Additionally, baseline IL‐6 levels correlated with progression in STIR signal over 12 months (*r* = 0.31, *p* = 0.02; Figure 4d), suggesting that early inflammatory activity, as reflected by IL‐6, is associated with greater progression of muscle edema. These findings support the role of IL‐6 not only as a marker of disease severity and activity but also as a potential biomarker of disease progression in FSHD1.

## 4 Discussion

FSHD1 is a complex genetic disorder characterized by progressive muscle weakness and atrophy, driven by both genetic and inflammatory factors. Inflammatory infiltrates, primarily CD4+ and CD8+ T cells, are consistently observed in muscle biopsies from FSHD1 patients, suggesting a significant immune‐mediated component to disease progression [7, 10]. Inflammation precedes muscle damage and fibrosis, exacerbating clinical symptoms and leading to irreversible structural and functional decline [31].

Given the central role of inflammation, several studies have evaluated cytokines as potential biomarkers for disease severity [10, 13, 14, 32, 33]. Among these cytokines, only serum IL‐6 has shown consistent promise [13, 14]. IL‐6, a pleiotropic cytokine involved in immune regulation, inflammation, and muscle repair through classical and trans‐signaling pathways [34, 35], is elevated in neuromuscular disorders like amyotrophic lateral sclerosis (ALS), Duchenne muscular dystrophy (DMD), and FSHD1, where it correlates with disease severity [13, 14, 15, 16].

This study reinforces IL‐6 as a key biomarker of disease severity, activity, and progression in FSHD1. Analysis of two independent cohorts (CTRN‐FSHD France and Cytokine FSHD) demonstrated a moderate correlation between serum IL‐6 levels and clinical severity measures (CSS, MMT sum score, 6MWT, MFM‐D1) over 18 months. Elevated IL‐6 levels also correlated with radiologic severity, specifically increased fat infiltration (T1 Sum Score) over 12 months, confirming IL‐6's potential as a disease severity indicator and aligned with prior findings [13, 14, 15, 16]. Our findings also reveal that elevated IL‐6 levels correlate with disease activity. Higher IL‐6 concentrations correlated positively with increased muscle edema associated with inflammation (STIR Sum Score), suggesting that IL‐6‐mediated inflammation plays a crucial role in the structural changes observed in FSHD1 muscles.

Muscle MRI provided a sensitive tool to quantify disease progression [10, 36, 37]. T1‐weighted imaging measured fatty infiltration, correlating strongly with clinical severity, while STIR imaging captured inflammatory edema reflecting disease activity [38]. Over the 12‐month period, both T1 and STIR Sum Scores increased significantly, corroborating the progressive nature of muscle degeneration and inflammatory activity in FSHD1 [11]. T1‐weighted imaging provided robust evidence of fatty infiltration across multiple muscle groups, with higher T1 Sum Scores correlating strongly with clinical severity markers such as the CSS and reduced functional performance (e.g., the 6MWT). These correlations persisted after 12 months, reinforcing the role of T1 imaging as a sensitive indicator of cumulative muscle damage. Moreover, since we used a semiquantitative scale for muscle MRI scoring, we were able to catch only the major MRI changes, those able to modify the Mercuri score [21], probably underestimating the power of a quantitative imaging technique in detecting even minimal disease progression. STIR imaging complemented these findings by capturing inflammatory activity through edema [38]. Elevated STIR hyperintense signals were associated with active disease processes, highlighting the inflammatory milieu as a critical driver of structural muscle changes. Notably, our study demonstrated a significant positive correlation between STIR and T1 Sum Scores, consistent with inflammatory activity contributing to structural changes [11, 18].

Importantly, longitudinal analysis revealed that changes in IL‐6 levels over time align with disease progression. Over 12 months, rising IL‐6 levels do not show any relationship with progression in clinical severity, but were associated with worsening MRI indicators of muscle composition, particularly fatty infiltration (T1 Sum Score) and edema (STIR Sum Score). The positive association between IL‐6 and T1 Sum Scores suggests that chronic inflammation, as reflected by elevated IL‐6, may contribute to fibro‐fatty muscle degeneration. Similarly, the correlation with STIR Sum Scores highlights IL‐6's involvement in active inflammatory processes, reinforcing its role in both the degenerative and inflammatory aspects of FSHD1. This is further reinforced by the positive correlation found between the levels of IL‐6 at baseline and the change over time in STIR Sum Scores, suggesting that higher initial IL‐6 levels are associated with greater progression of muscle inflammation. Since an increasing STIR hyperintense signal reflects worsening disease activity [8, 11, 38], these findings support the notion that IL‐6 is not a secondary consequence of muscle degeneration but may actively participate in the disease's pathophysiology, making it a promising biomarker for tracking disease activity and progression.

While our study provides evidence that serum IL‐6 levels effectively reflect disease severity and activity in FSHD1, certain methodological limitations should be acknowledged. The MRI analyses were based on semi‐quantitative scoring, which, although widely used in neuromuscular research [39, 40], has, by nature, a degree of subjectivity. To minimize bias, all images were scored by two experienced radiologists who were blinded to the data and acquisition time; discrepancies were resolved by consensus, and inter‐rater agreement exceeded 80%. The monocentric nature of the study can also be a limitation to results generalizability, but the analysis was made from two independent but harmonized cohorts of 30 patients, thus enhancing consistency and results robustness. Finally, although a formal whole‐body MRI protocol was used to assess 94 muscles per patient, we recognize the value of quantitative MRI and multicentric validation in future studies to strengthen generalizability and reduce interpretative variability.

Despite the generally slow rate of progression observed in FSHD, measurable deterioration over a one‐year period has been consistently documented, particularly when using quantitative strength assessments and enrolling patients with established weakness. Even though annual changes are typically modest, subtle but significant declines in muscle strength or ability to stand and transfer can be detected within a 12‐month interval [41, 42, 43]. In our studies, we specifically enrolled patients who already had symptomatic lower‐limb weakness (at least one lower‐limb muscle with MMT ≥ 4); from both the literature and clinical experience, such patients are more prone to show progression over a year [44]. Notably, change over 12 months in IL‐6 exhibited stronger correlations with change in MRI‐based markers than with change in conventional functional measures, such as the 6MWT or MFM‐D1. The lack of correlation with the change in other clinical metrics over time may indicate that these metrics are less sensitive to early pathological changes or that they reflect different aspects of disease progression. For instance, measures like the MMT and MFM‐D1 might not capture subtle changes in muscle composition and inflammation that occur early in the disease process. These functional tests often require more significant muscle deterioration before changes become apparent [27, 45], whereas IL‐6 levels may increase in response to early inflammatory processes that precede noticeable functional decline.

Interestingly, while serum IL‐6 levels showed a significant rise from baseline to 12 months, a subsequent decrease was observed at the 18‐month follow‐up, although levels remained elevated compared to baseline. This suggests that IL‐6 may reflect transient peaks of inflammatory activity rather than a linear accumulation over time. The decline at 18 months occurred despite continued clinical deterioration, indicating that IL‐6 may act upstream in the disease cascade, associated with increased inflammation that precedes structural muscle damage and functional loss. It is plausible that IL‐6 elevation reflects an active inflammatory phase (associated with STIR hyperintense signaling on muscle MRI), which then initiates degenerative processes (e.g., fatty infiltration, fibrosis) that manifest clinically with some delay. This hypothesis is supported by previously reported results showing that STIR positive muscle progresses faster toward fatty replacement and muscle destruction [12, 29, 30]. Additionally, the observed decline in IL‐6 at 18 months may result from biological feedback mechanisms regulating chronic inflammation [46] or reflect inter‐individual variability in disease dynamics. Overall, these findings support the concept of IL‐6 as a dynamic biomarker that captures inflammatory shifts in FSHD1.

Previous studies suggested that muscular dystrophies progression does not necessarily occur linearly, but rather through episodic inflammatory and degenerative events driving muscle deterioration [47, 48, 49]. It is also possible that the relative progression observed in our cohort may reflect transient peaks of disease activity characterized by elevated inflammation and dynamic muscle remodeling processes. Supporting this hypothesis, we found that baseline IL‐6 levels correlated with subsequent increases in STIR Sum Score, associated with muscle edema and active inflammation. It is thus plausible that our patient selection criteria captured individuals during an active phase of the disease, contributing to the measurable progression documented in this relatively short follow‐up period.

From a pathophysiological perspective, IL‐6 likely plays a dual role in FSHD1, contributing to both inflammation‐driven muscle damage and compensatory muscle repair [35, 50]. IL‐6 is known to mediate immune activation through trans‐signaling pathways, perpetuating chronic inflammation and tissue degeneration [51]. At the same time, IL‐6 can promote muscle regeneration—a paradoxical function observed in other neuromuscular disorders [15, 16, 52, 53]. This duality underscores the need for further mechanistic studies to determine whether IL‐6 is a driver of pathology or a compensatory response to muscle injury in FSHD1. Further, longer follow‐up studies are necessary to determine the consistency of IL‐6 levels over extended periods and their correlation with long‐term disease outcomes. Such data are crucial for validating IL‐6 as a reliable biomarker for monitoring disease progression and therapeutic efficacy. A key next step is to evaluate the test–retest reliability of circulating IL‐6 levels in FSHD, as IL‐6 demonstrates circadian oscillations and can be influenced by transient factors like physical activity or infection [54, 55]. Although our study minimized variability by using fasting morning samples, dedicated investigations sampling across different times and days are needed to confirm biomarker stability.

The association between IL‐6 and disease progression also has therapeutic implications. In the context of FSHD1, therapies aimed at blocking IL‐6 signaling could potentially reduce inflammatory damage, limit muscle fibrosis, and slow disease progression. This hypothesis is currently being explored in a Phase 2 clinical trial using Satralizumab, an anti‐IL‐6 receptor monoclonal antibody (NCT06222827). Satralizumab, by antagonizing IL‐6R downstream signaling, holds promise in mitigating inflammation and potentially curtailing fibrofatty degeneration in FSHD1. However, it remains to be seen whether IL‐6 inhibition will have a significant impact on disease progression or if IL‐6 is primarily a disease marker rather than a modifiable pathway. Indeed, reductions in IL‐6 may indicate either a true therapeutic effect on chronic inflammatory pathways or the natural resolution of episodic STIR‐positive muscle edema. Longitudinal MRI studies have shown that muscles with STIR hyperintensity tend to progress more rapidly to fat replacement, whereas STIR‐negative muscles often remain stable over time [11, 12]. For this reason, IL‐6 trends should be interpreted alongside imaging and functional outcomes to help distinguish genuine treatment effects from natural disease fluctuations.

In conclusion, this study confirms IL‐6 as a relevant biomarker for disease severity in FSHD1 and highlights its role as a potential biomarker of disease activity and progression. Our results demonstrate that higher IL‐6 levels are associated with more severe clinical symptoms, greater muscle fat infiltration, and increased inflammatory edema, highlighting its role in both structural and inflammatory disease processes. Longitudinal data further confirm that IL‐6 levels track disease progression, making it a valuable tool for monitoring FSHD1 over time. These findings not only enhance our understanding of FSHD1 progression but also pave the way for improved monitoring and treatment strategies, addressing an urgent need in the clinical landscape of this devastating disorder.

## Author Contributions

N.G., and S.S. contributed to the study's conception and design. E.M., A.P., L.V., M.C., A.E., J.G., M.G., F.T., L.J.P. and S.S. contributed to the data acquisition. J.P., E.M., S.D., A.S., N.G. and S.S. contributed to data analysis. J.P. and S.S. wrote the manuscript and prepared the Figs.

## Ethics Statement

The Cytokines FSHD study (NCT04694456) and the CTRN‐FSHD France study (NCT04038138) study comply with the Data Protection Act n° 78‐17 of January 6, 1978, as amended, and with Regulation (EU) 2016/679 of the European Parliament and of the Council of April 27, 2016, applicable from May 25, 2018 (General Data Protection Regulation). All the participants signed an informed consent form prior to any study‐related procedures. The Cytokines FSHD study (NCT04694456) and the CTRN‐FSHD France study (NCT04038138) were approved by the ethics committee Sud Méditerranée V on the 11th of April 2017 (reference 16.054) and the 09th of May 2019 (reference 19.03.12.39900), respectively.

## Consent

The authors have nothing to report.

## Conflicts of Interest

Prof. S. SACCONI served as a consultant for Fulcrum Therapeutics and Hoffmann‐La Roche. She is also the PI of the ReInForce study (NCT06222827). All other authors report no competing interests.

## Supporting information


**Figure S1:** Representative MRI images of STIR edema and T1‐weighted fat infiltration.
**Figure S2:** Correlation between T1 Sum Score and clinical, and functional score at baseline.
**Figure S3:** Correlation between T1 Sum Score and clinical, and functional score at the 12‐month visit.
**Figure S4:** Correlation between STIR Sum Score and clinical, and functional score at baseline.
**Figure S5:** Correlation between STIR Sum Score and clinical, and functional score at the 12‐month visit.
**Figure S6:** Comparison of T1 Sum score, STIR Sum Score and IL‐6 levels between Baseline, the 12‐ and 18‐month visits.
**Table S1:** List of the evaluated muscles.
**Table S2:** Details of the muscle's classification based on the percentage of fat infiltration and the presence of muscle edema.
**Table S3:** Schedule of assessment for the Cytokine FSHD and the CTRN‐FSHD France studies.
**Table S4:** Correlations between T1 or STIR Sum scores and clinical scores adjusted for age at Baseline and 12 months.
**Table S5:** Correlations between IL‐6 levels, T1 Sum Score, STIR Sum score, and clinical scores adjusted for age at Baseline, 12 and 18 months.
**Table S6:** Aggregated data.

## Data Availability

The data that support the findings of this study are available from the corresponding author on reasonable request. A fully de‐identified, aggregated version of the dataset (variables and summary statistics) is provided as Table S6.
