# Supplementary material for: Interleukin‐6 as a Key Biomarker in Facioscapulohumeral Dystrophy: Evidence From Longitudinal Analyses
Source: Ann Clin Transl Neurol. 2025 Oct 7;13(2):310–23. doi: 10.1002/acn3.70210 (PMC12883698; doi:10.1002/acn3.70210)
Supplement: Supplementary file 1 — Figure S1: Representative MRI images of STIR edema and T1‐weighted fat infiltration. Figure S2: Correlation between T1 Sum Score and clinical, and functional score at baseline. Figure S3: Correlation between T1 Sum Score and clinical, and functional score at the 12‐month visit. Figure S4: Correlation between STIR Sum Score and clinical, and functional score at baseline. Figure S5: Correlation between STIR Sum Score and clinical, and functional score at the 12‐month visit. Figure S6: Comparison of T1 Sum score, STIR Sum Score and IL‐6 levels between Baseline, the 12‐ and 18‐month visits. Table S1: List of the evaluated muscles. Table S2: Details of the muscle's classification based on the percentage of fat infiltration and the presence of muscle edema. Table S3: Schedule of assessment for the Cytokine FSHD and the CTRN‐FSHD France studies. Table S4: Correlations between T1 or STIR Sum scores and clinical scores adjusted for age at Baseline and 12 months. Table S5: Correlations between IL‐6 levels, T1 Sum Score, STIR Sum score, and clinical scores adjusted for age at Baseline, 12 and 18 months. Table S6: Aggregated data. [file ACN3-13-310-s001.docx]

**SUPPLEMENTARY TABLES**

**Supplementary Table 1: List of the evaluated muscles**

| **Axial Muscles** | Sternocleidomastoideus | **Lower Limb Muscles** | Ileo Psoas |
| --- | --- | --- | --- |
|  | Scaleni |  | Gluteus Maximus |
|  | Paraspinous (Cervical) |  | Gluteus Medius |
|  | Paraspinous (Thoracic) |  | Gluteus Minimus |
|  | Iliocostalis Lomborum |  | Tensor Fasciae Latae |
|  | Quadratus Lomborum |  | Sartorius |
|  | Rectus Abdominis |  | Gracilis |
|  |  |  | Rectus Femoris |
| **Upper Limb Muscles** | Levator Scapulae |  | Vastus Intermedius |
|  | Trapezius |  | Vastus Medialis |
|  | Subscapularis |  | Vastus Lateralis |
|  | Supraspinatus |  | Biceps Femoris Caput Longum |
|  | Infraspinatus/ Teres Minor |  | Biceps Femoris Caput Breve |
|  | Serratus anterior |  | Semitendinosus |
|  | Rhomboidei |  | Semimembranosus |
|  | Deltoideus |  | Adductor Magnus |
|  | Latissimus Dorsi / Teres Major |  | Adductor Longus |
|  | Pectoralis Major |  | Adductor Brevis |
|  | Pectoralis Minor |  | Pectineus |
|  | Biceps Brachii* |  | Obturatorius Internus |
|  | Coracobrachialis* |  | Popliteus |
|  | Brachialis* |  | Gastrocnemius Medialis |
|  | Triceps Brachii |  | Gastrocnemius Lateralis |
|  |  |  | Soleus |
|  |  |  | Tibialis Anterior ** |
|  |  |  | Extensor Digitorum Longus ** |
|  |  |  | Extensor Hallucis Longus ** |
|  |  |  | Peroneus Longus |
|  |  |  | Tibialis Posterior |

94 muscles (47 left and 47 right) were evaluated to calculate the T1 Score and the STIR Score

* / **: Muscles were considered as one entity because of the difficulty to distinguish

**Supplementary Table 2: Details of the muscle’s classification based on the percentage of fat infiltration and the presence of muscle edema**

| **T1-weighted Images (Fat Infiltration)** | | | | |
| --- | --- | --- | --- | --- |
| Class | Description | Fat % range | | Reference value |
| 0 | Normal muscle | 0% | | 0.0 |
| 1 | Mild hyperintensity (<15%) | ]0–14.99%] | | 7.5% |
| 2 | Moderate hyperintensity (<50%) | [15–49.99%] | | 32.5% |
| 3 | Severe hyperintensity (>50%) | [50–99.99%] | | 75% |
| 4 | Complete fat replacement or atrophy | 100% | | 100% |
|  |  |  | |  |
| **STIR Images (Edema)** | |  |  | |
| Class | Description |  | |  |
| 0 | No hyperintensity (no edema) |  | |  |
| 1 | Hyperintense signal indicating edema |  | |  |

**Supplementary Table 3: Schedule of assessment for the Cytokine FSHD and the CTRN-FSHD France studies**

| Cytokine FSHD (NCT04694456) | | | | CTRN-FSHD France (NCT04038138) | | | |
| --- | --- | --- | --- | --- | --- | --- | --- |
| Visits | **Visit 1** | **Visit 2** | **Visit 3** | **Visits** | **Visit 1** | **Visit 2** | **Visit 3** |
| Time (Months) | 0 | 12 ± 1 | 18 ± 1 | **Time (Months)** | 0 | 12 ± 1 | 18 ± 1 |
| Informed consent | X |  |  | **Informed consent** | X |  |  |
| Confirm elligibility | X |  |  | **Confirm elligibility** | X |  |  |
| Urine Pregnancy Test | X |  |  | **Urine Pregnancy Test** | X |  |  |
| History and Physical Exam | X |  |  | **History and Physical Exam** | X |  |  |
| MRI | X | X |  | **MRI** | X | X | X |
| Manual Muscle Testing (MMT) | X | X | X | **Manual Muscule Testing (MMT)** | X | X | X |
| 6 Minute Walk Test (6MWT) | X | X | X | **6MWT (derived from FSHD-COM)** | X | X | X |
| Motor Function Measure 32 items (MFM32) | X | X | X | **MFM Domain 1** | X | X | X |
| Clinical severity scale (CSS) | X | X | X | **Clinical Severity Scale (CSS)** | X | X | X |
| Blood for Serum Extraction | X | X | X | **Blood for Serum Extraction** | X | X | X |
|  |  |  |  | **FSHD-COM** | X | X | X |
|  |  |  |  | **Bedside spirometry** | X | X | X |
|  |  |  |  | **DEXA** | X |  | X |
|  |  |  |  | **Quantitative Muscular Testing** | X | X | X |
|  |  |  |  | **Fall/Exercise Questionnaire** | X | X | X |
|  |  |  |  | **PROs: PROMIS57, UEFI, and FDI physical score** | X | X | X |

The items highlighted are the common items between the two studies that were analyzed.

**Supplementary Table 4: Correlations between T1 or STIR Sum scores and clinical scores adjusted for age at Baseline and 12 months.**

| T1 Sum Score | | | | |
| --- | --- | --- | --- | --- |
|  | Baseline | | 12 months | |
|  | r | p-value | r | p-value |
| Age-corrected CSS | 0.64 | <0.0001 | 0.71 | <0.0001 |
| MMT | -0.79 | <0.0001 | -0.78 | <0.0001 |
| 6MWT | -0.63 | <0.0001 | -0.60 | <0.0001 |
| MFM-D1 | -0.84 | <0.0001 | -0.79 | <0.0001 |
|  |  |  |  |  |
| STIR Sum Score | | | | |
|  | Baseline | | 12 months | |
|  | r | p-value | r | p-value |
| Age-corrected CSS | 0.2 | 0.05 | 0.48 | 0.003 |
| MMT | - | NS | -0.48 | 0.0001 |
| 6MWT | - | NS | - | NS |
| MFM-D1 | -0.36 | 0.02 | -0.39 | 0.0019 |
| T1 Sum Score | 0.36 | 0.008 | 0.64 | <0.0001 |

Spearman’s or Pearson’s correlation coefficient (r) and p-value for the T1 or STIR Sum scores, and clinical scores at Baseline and the 12-month visit.

**Supplementary Table 5: Correlations between IL-6 levels, T1 Sum Score, STIR Sum score, and clinical scores adjusted for age at Baseline, 12 and 18 months.**

| Log(IL-6) | | | | | | |
| --- | --- | --- | --- | --- | --- | --- |
|  | Baseline | | 12 months | | 18 months | |
|  | r | p-value | r | p-value | r | p-value |
| Age-corrected CSS | - | NS | - | NS | 0.38 | 0.006 |
| MMT | -0.3 | 0.01 | -0.26 | 0.02 | -0.23 | 0.04 |
| 6MWT | -0.35 | 0.012 | -0.30 | 0.029 | -0.39 | 0.004 |
| MFM-D1 | -0.36 | 0.008 | -0.23 | 0.04 | -0.38 | 0.004 |
| T1 Sum Score | 0.41 | 0.002 | 0.30 | 0.014 | - | - |
| STIR Sum Score | 0.26 | 0.029 | 0.31 | 0.013 | - | - |

Spearman’s or Pearson’s correlation coefficient (r) and p-value for the serum IL-6 levels, and T1 Sum score, STIR Sum score, and clinical scores at Baseline, the 12- and 18-month visit.

**Supplementary Table 6: Aggregated data.**

| Variable | Mean | SD | Median | Min | Max |
| --- | --- | --- | --- | --- | --- |
| RU number | 6.62 | 1.62 | 7 | 3 | 10 |
| Age at disease onset | 32.77 | 16.07 | 30 | 10 | 70 |
| Age at Baseline | 53.29 | 15.02 | 58 | 22 | 74 |
| Disease duration | 20.52 | 14.38 | 18 | 0 | 56 |
| Age at 12 months | 54.29 | 15.02 | 59 | 23 | 75 |
| Disease duration | 21.52 | 14.38 | 19 | 1 | 57 |
| Age at 18 months | 54.42 | 15.10 | 59.5 | 23 | 75 |
| Disease duration | 21.65 | 14.46 | 19.5 | 1 | 57 |
| CSS M0 | 5.44 | 2.12 | 6 | 2 | 8 |
| CSS M12 | 6.17 | 1.69 | 6 | 2 | 9 |
| CSS M18 | 6.08 | 1.76 | 6 | 2 | 9 |
| Age corrected CSS M0 | 109.13 | 52.06 | 103.45 | 28.17 | 269.23 |
| Age corrected CSS M12 | 122.13 | 48.78 | 111.11 | 34.48 | 296.30 |
| Age corrected CSS M18 | 119.16 | 48.46 | 108.70 | 34.48 | 296.30 |
| MMT sum score M0 | 114.65 | 17.61 | 117.51 | 69.33 | 140 |
| MMT sum score M12 | 110.86 | 18.63 | 112.18 | 71.33 | 138.04 |
| MMT sum score M18 | 110.96 | 19.87 | 114.20 | 65.28 | 139.72 |
| 6MWT M0 | 464.73 | 168.5 | 496.50 | 129 | 750 |
| 6MWT M12 | 397.62 | 173.6 | 435 | 72 | 740 |
| 6MWT M18 | 411.35 | 179.8 | 449.5 | 72 | 741 |
| MFM - D1 M0 | 31.06 | 8.85 | 34 | 7 | 39 |
| MFM - D1 M12 | 28.31 | 9.75 | 32.5 | 5 | 39 |
| MFM - D1 M18 | 28.63 | 10.03 | 31.5 | 4 | 39 |
| T1 Sum Score M0 | 0.20 | 0.17 | 0.18 | 0.00 | 0.58 |
| T1 Sum Score M12 | 0.26 | 0.20 | 0.20 | 0.00 | 0.69 |
| STIR Sum Score M0 | 0.07 | 0.06 | 0.06 | 0.00 | 0.22 |
| STIR Sum Score M12 | 0.14 | 0.12 | 0.14 | 0.00 | 0.59 |
| IL6 M0 (pg/ml) | 53.93 | 42.74 | 44.94 | 8.03 | 283.82 |
| IL6 M12 (pg/ml) | 125.18 | 117.1 | 100.39 | 14.45 | 707.84 |
| IL6 M18 (pg/ml) | 88.81 | 67.9 | 71.58 | 13.80 | 364.22 |
| log(IL6-M0) | 1.63 | 0.30 | 1.65 | 0.90 | 2.45 |
| log(IL6-M12) | 1.97 | 0.33 | 2.00 | 1.16 | 2.85 |
| log(IL6-M18) | 1.85 | 0.19 | 1.85 | 1.14 | 2.56 |

**SUPPLEMENTARY FIGURES**

**
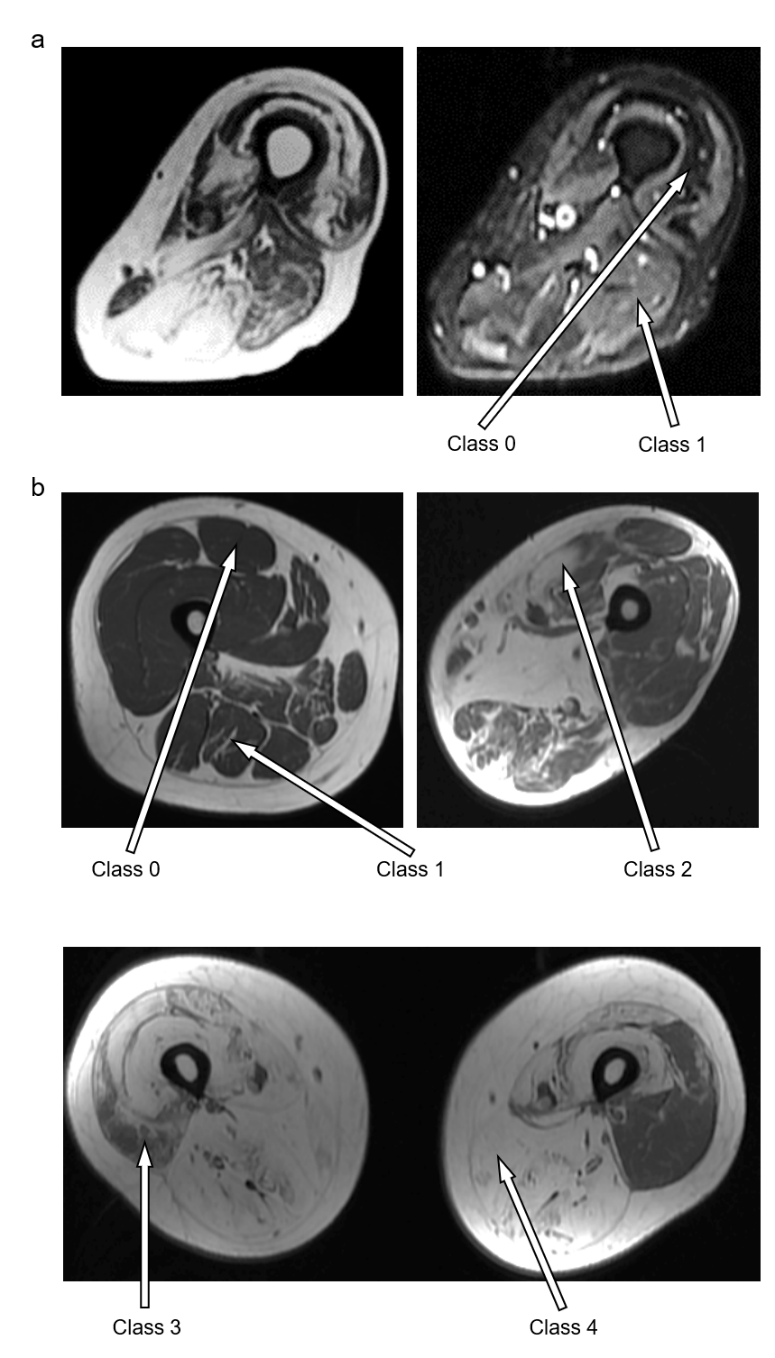
**

**Supplementary figure 1: Representative MRI images of STIR edema and T1-weighted fat infiltration.**

a) Axial STIR sequences of the thigh muscles are shown on the right-hand side, with corresponding T1- weighted images on the left. Representative images for STIR+ (class 1, *biceps femoris*) and STIR- (class 0, *vastus intermedius*) muscles.

b) Axial T1-weigthed sequences of the thigh muscles showed the 5 different classes of fat infiltration in muscle, according to the Mercury scoring. Representative image of the class 0 (*Rectus Femoris*), class 1 (*Semitendinosus*), class 2 (*Vastus medialis*), class 3 (*Vastus lateralis*) and class 4 (*Biceps femoris*).


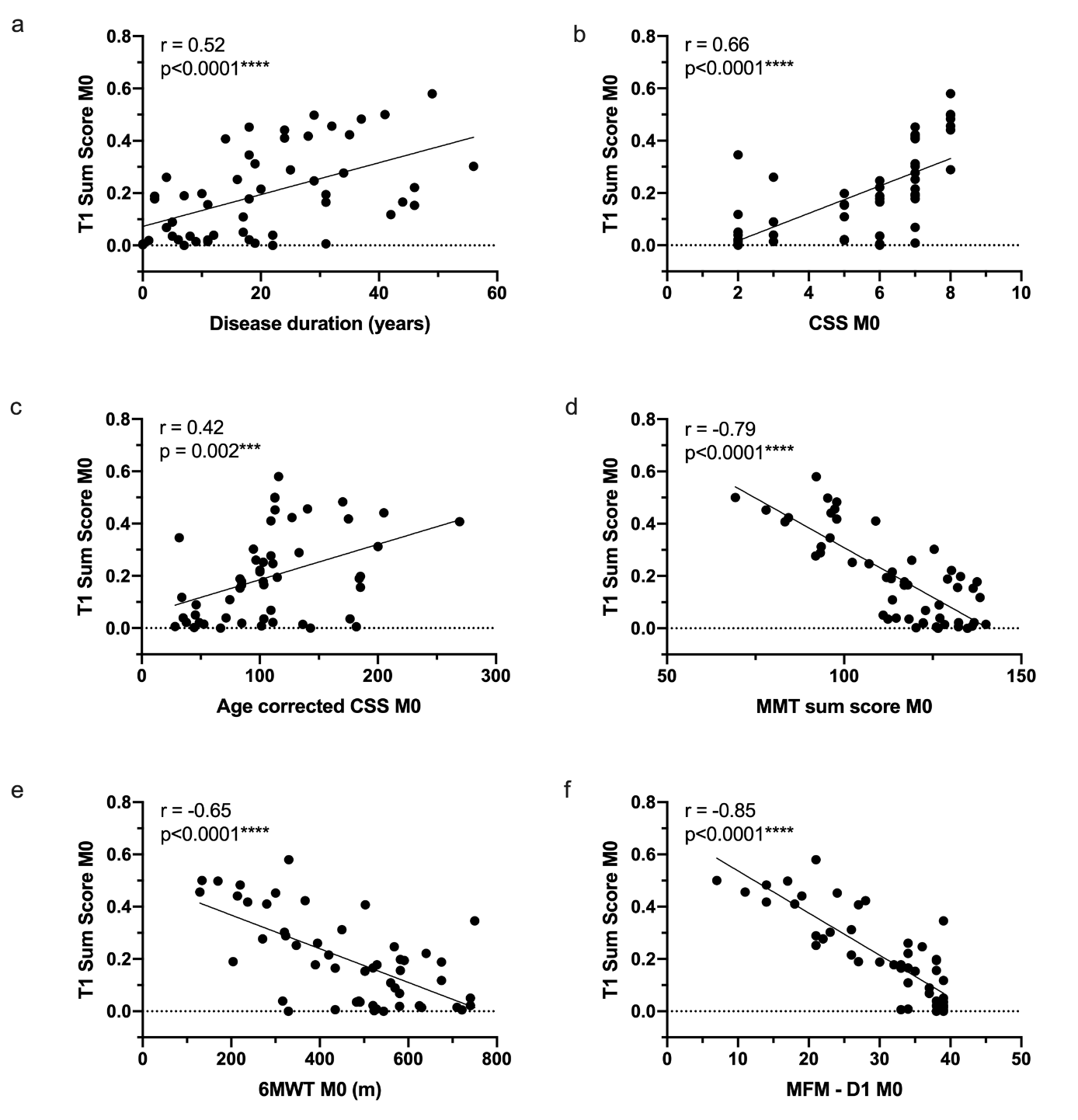


**Supplementary figure 2: Correlation between T1 Sum Score and clinical, and functional score at baseline.**

Scatter plot representations of T1 Sum Score versus disease Duration (a), clinical severity score (CSS; b), age-corrected CSS (c), Manual Muscle Testing (MMT) sum score (d), 6 Minute Walk Test (6MWT; e), Domain 1 of the Motor Function Measure (MFM-D1; f), and the STIR Sum Score (g), at baseline. Spearman R coefficients and p-values are indicated.


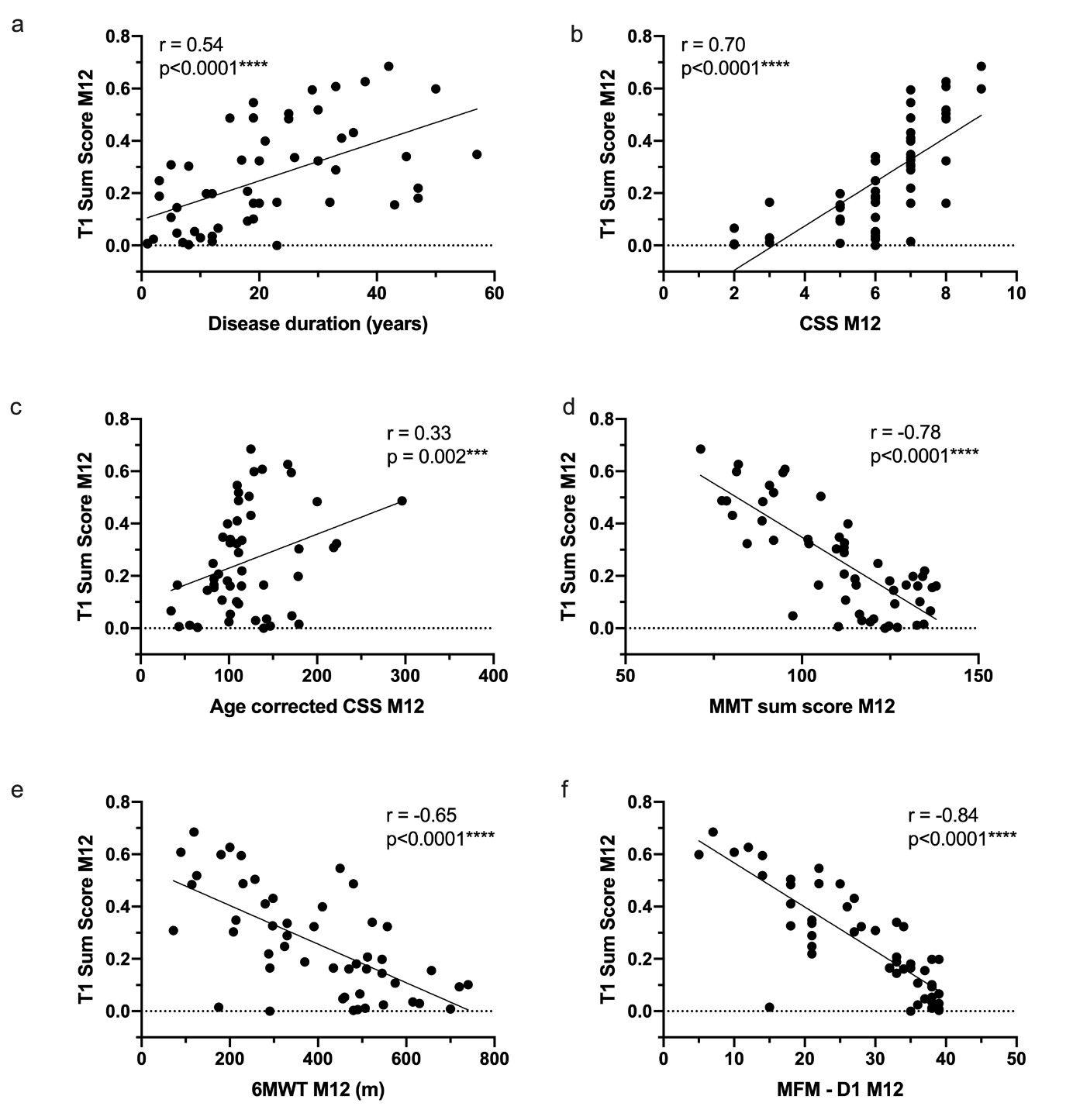


**Supplementary figure 3: Correlation between T1 Sum Score and clinical, and functional score at the 12-month visit.**

Scatter plot representations of T1 Sum Score versus disease Duration (a), clinical severity score (CSS; b), age-corrected CSS (c), Manual Muscle Testing (MMT) sum score (d), 6 Minute Walk Test (6MWT; e), Domain 1 of the Motor Function Measure (MFM-D1; f), and the STIR Sum Score (g), at the 12-month visit. Spearman R coefficients and p-values are indicated.


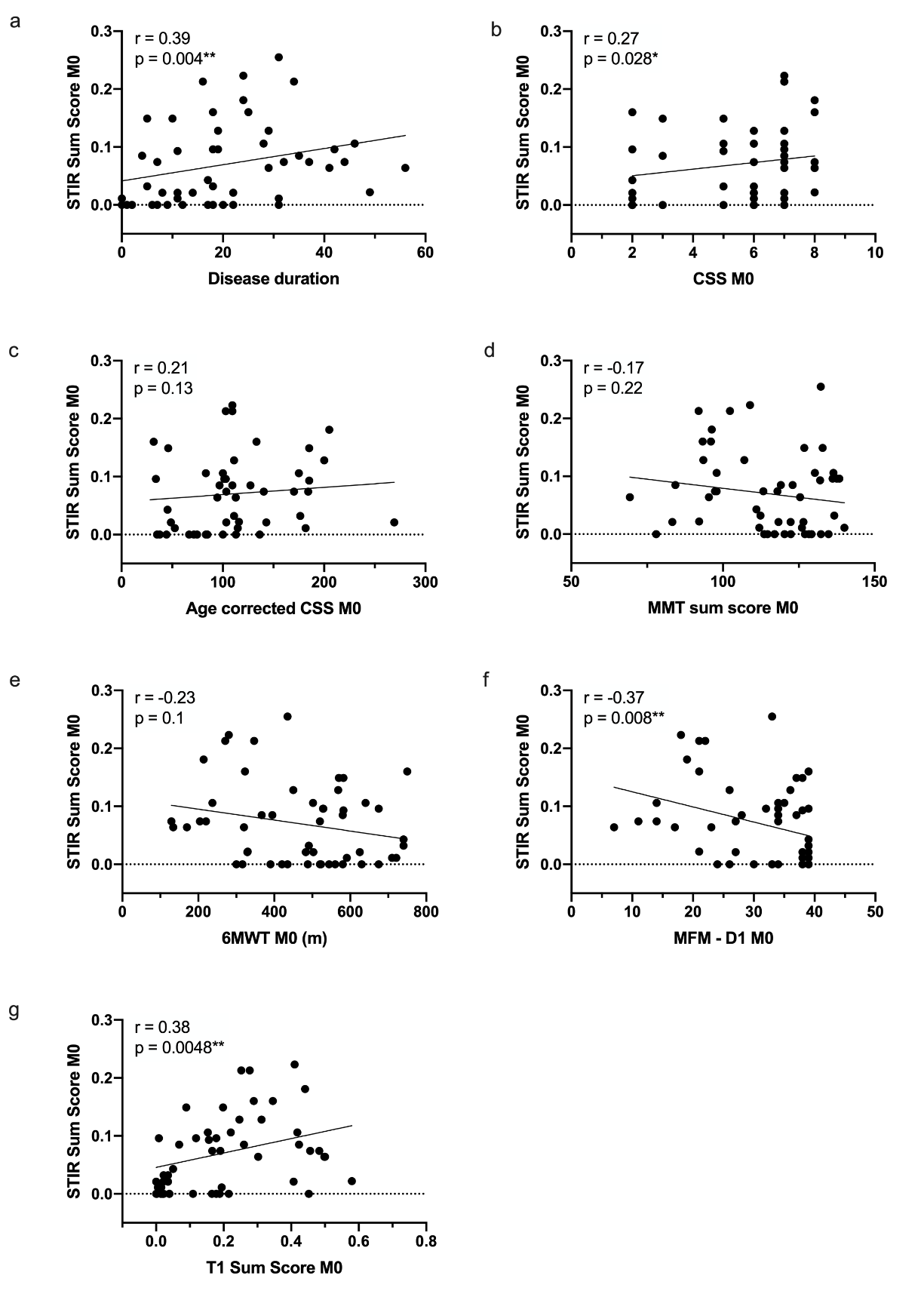


**Supplementary figure 4: Correlation between STIR Sum Score and clinical, and functional score at baseline.**

Scatter plot representations of T1 Sum Score versus disease Duration (a), clinical severity score (CSS; b), age-corrected CSS (c), Manual Muscle Testing (MMT) sum score (d), 6 Minute Walk Test (6MWT; e), Domain 1 of the Motor Function Measure (MFM-D1; f), and the T1 Sum Score (g), at baseline. Spearman R coefficients and p-values are indicated.


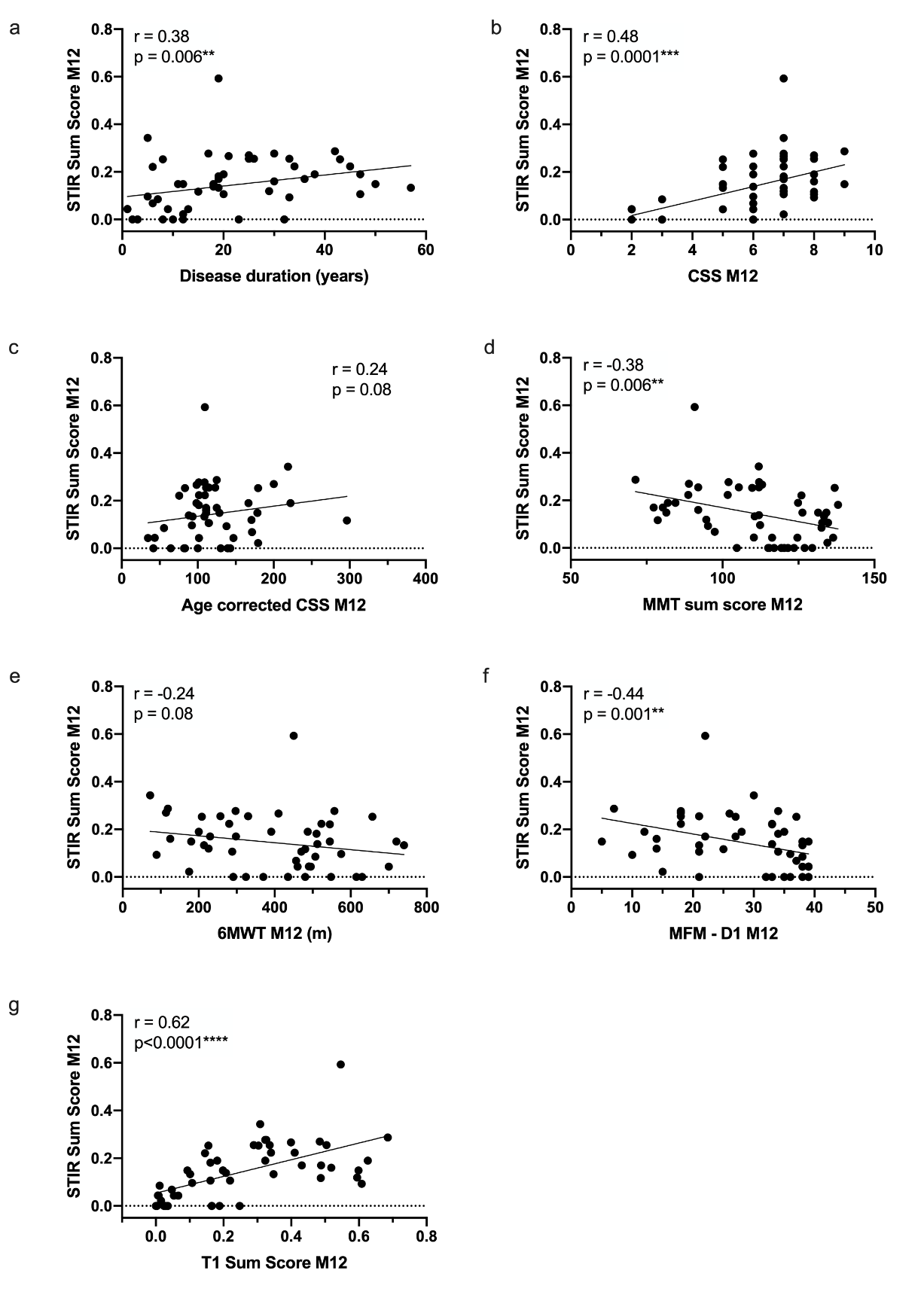


**Supplementary figure 5: Correlation between STIR Sum Score and clinical, and functional score at the 12-month visit.**

Scatter plot representations of T1 Sum Score versus disease Duration (a), clinical severity score (CSS; b), age-corrected CSS (c), Manual Muscle Testing (MMT) sum score (d), 6 Minute Walk Test (6MWT; e), Domain 1 of the Motor Function Measure (MFM-D1; f), and the T1 Sum Score (g), at the 12-month visit. Spearman R coefficients and p-values are indicated


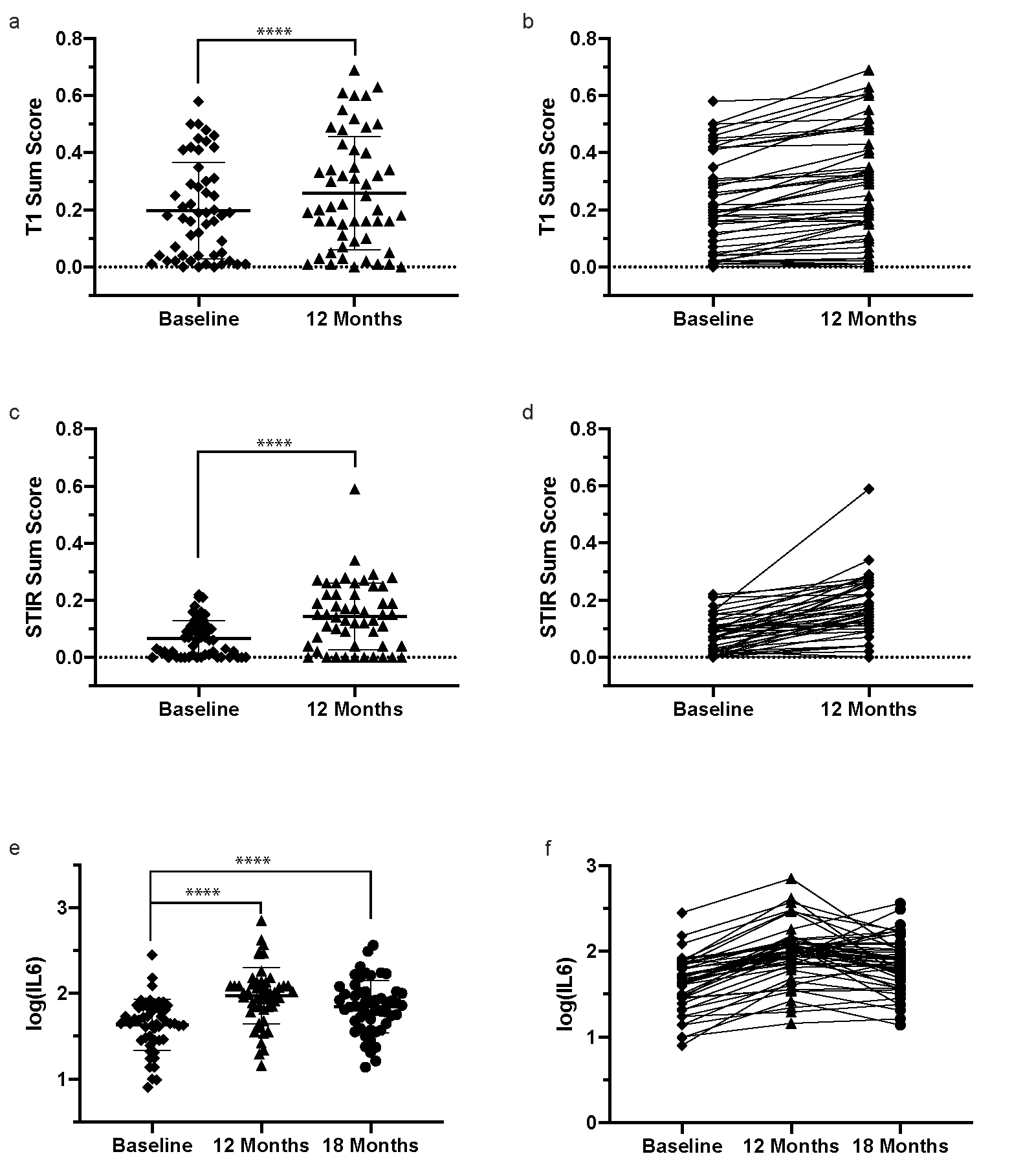


**Supplementary figure 6: Comparison of T1 Sum score, STIR Sum Score and IL-6 levels between Baseline, the 12- and 18-month visits.**

Scatter plot representations of (a) T1 Sum Score at Baseline and the 12-month visit and (b) individual’s evolution over time; (c) STIR Sum Score at Baseline and the 12-month visit and (d) individual’s evolution over time; and (e) IL-6 levels at Baseline, the 12- and 18-month visit and (f) individual’s evolution over time. A significant difference between visits was obtained (p< 0.0001)
